# Association between adherence to the Mediterranean Diet and pancreatic cancer risk: a systematic review and meta-analysis

**DOI:** 10.3389/fnut.2026.1925019

**Published:** 2026-09-04

**Authors:** Yuxin Zhang, Mingyu Du, Luyang Li, Yanan Xing

**Affiliations:** 1 Department of Gastrointestinal Surgery, The First College of Clinical Medical Science, Yichang Central People’s Hospital, China Three Gorges University, Yichang, China; 2 China Three Gorges University, Yichang, Hubei, China

**Keywords:** Hazard Ratio, Mediterranean Diet, meta-analysis, pancreatic cancer, prevention

## Abstract

**Objectives:**

Pancreatic cancer (PC) is one of the most lethal malignancies with a poor prognosis. Although the Mediterranean Diet (MedDiet) is hypothesized to be inversely associated with PC risk, epidemiological findings remain inconsistent. This meta-analysis aims to quantitatively evaluate the association between Mediterranean Diet adherence and the risk of developing pancreatic cancer by synthesizing the most recent evidence.

**Methods:**

A systematic search was performed across PubMed, Web of Science, Embase, and the Cochrane Library for observational studies investigating MedDiet and PC risk. Data were synthesized using both continuous and categorical variable analyses. Given the anticipated heterogeneity, a random-effects model was applied to pool the effect estimates.

**Results:**

Fourteen studies (12 cohorts and 2 case-control studies) were eligible for inclusion. In the continuous analysis, a higher MedDiet adherence score was associated with a lower risk of PC (pooled relative effect estimates per one-unit increase within the respective scoring system = 0.96, 95% CI: 0.94–0.97; I^2^ = 58.8%). In the categorical analysis, participants in the highest adherence group had a 10% lower risk of PC compared to those in the lowest group (pooled relative effect estimates = 0.90, 95% CI: 0.86–0.94, I^2^ = 72.4%). Subgroup analyses: The inverse association was consistently observed in both males and females. Sensitivity analyses indicated that the pooled relative effect estimates remained consistently below 1.0 (ranging from 0.75 to 0.98) after omitting individual studies. Nevertheless, Egger’s test suggested potential publication bias only for the categorical variables, but not for the continuous variables.

**Conclusion:**

This meta-analysis suggests an inverse association between higher adherence to the MedDiet and pancreatic cancer risk. However, the observational nature of the available evidence limits causal inference. Further well-designed prospective studies and dietary intervention trials are warranted to confirm this association and clarify its potential implications for pancreatic cancer prevention.

**Systematic review registration:**

https://www.crd.york.ac.uk/prospero/, identifier CRD420261459967.

## 1 Introduction

Pancreatic cancer is a highly lethal malignancy, characterized by aggressive tumor biology and non-specific early symptoms (1). According to 2024 data from the American Cancer Society, although the 5-year survival rate for pancreatic cancer has marginally increased to 13%, it remains the lowest among all major cancer types (2). While the etiology of pancreatic cancer is not yet fully elucidated, several modifiable risk factors have been identified, including diabetes, smoking, obesity, alcohol consumption (3, 4), and dietary factors (5).

In this context, dietary patterns are increasingly recognized as critical mediators of disease risk, particularly through their influence on immune modulation and the maintenance of gut microbiota diversity and homeostasis (6). Among various dietary regimes, plant-based diets—such as vegan, semi-vegetarian, and Mediterranean patterns—have been associated with a reduced risk of pancreatic cancer (7). The MedDiet is characterized by a high consumption of fruits, vegetables, whole grains, legumes, nuts, and olive oil, complemented by moderate intake of fish and poultry. This pattern is prevalent in southern European nations, including Greece, Spain, France, and southern Italy. Metabolites associated with the MedDiet, such as omega-3 fatty acids, tyrosine, and citrate, have shown significant inverse correlations with overall cancer risk (8). Furthermore, strong adherence to the MedDiet has been linked to a decreased incidence of cardiometabolic diseases, various cancers, and other chronic conditions (9). However, the epidemiological evidence regarding the association between MedDiet adherence and pancreatic cancer risk remains inconsistent. For instance, while Julián-Serrano et al. reported a significant protective effect in a U.S. cohort (10), Molina-Montes et al. found no significant association in a multicenter study spanning ten European countries (11). Although a previous meta-analysis suggested that high adherence to the MedDiet might reduce pancreatic cancer risk (12), recent high-quality studies have produced conflicting results, reigniting scientific debate (13, 14).

Given the significant global burden of pancreatic cancer and the urgent need for clarifying effective risk-related strategies, this study aims to update the existing evidence by conducting a meta-analysis that integrates data established previously with new studies published in the last 3 years. By systematically synthesizing all available evidence, this study seeks to clarify the association of the MedDiet with pancreatic cancer risk, thereby providing a scientific basis for public health recommendations and targeted intervention strategies.

## 2 Materials and methods

### 2.1 Search strategies and data sources

This systematic review and meta-analysis was conducted in accordance with the Preferred Reporting Items for Systematic Reviews and Meta-Analyses (PRISMA) 2020 guidelines (15). The review protocol was retrospectively registered in PROSPERO after completion of the main analyses (Registration ID: CRD420261459967). A structured electronic search was performed across PubMed, Embase, Web of Science, and the Cochrane Library, covering the period from database inception to 24 July 2026.

The search strategy employed a combination of Medical Subject Headings (MeSH) and free-text terms, connected using the Boolean operators “AND” and “OR.” Keywords included “Mediterranean Diet,” “pancreatic cancer,” and their respective synonyms. The specific search strategy, initially developed for PubMed and adapted for other databases, is detailed in Table 1. Additionally, reference lists of relevant systematic reviews and meta-analyses published since January 2023 were manually screened to identify any potentially missed articles.

**TABLE 1:** Search strategy.

Database	Syntax
Pubmed	(“Pancreatic Neoplasms”[Mesh] OR “pancreatic cancer*”[Title/Abstract] OR “pancreas cancer*”[Title/Abstract] OR “pancreatic carcinoma*”[Title/Abstract] OR “pancreatic neoplasm*”[Title/Abstract] OR “pancreatic tumor*”[Title/Abstract] OR “pancreatic tumor*”[Title/Abstract] OR “pancreatic adenocarcinoma*”[Title/Abstract] OR “PDAC”[Title/Abstract]) AND (“Diet, Mediterranean”[Mesh] OR “Mediterranean diet*”[Title/Abstract] OR “Mediterranean dietary”[Title/Abstract] OR “Mediterranean pattern*”[Title/Abstract] OR “Mediterranean-style*”[Title/Abstract] OR “Mediterranean-like*”[Title/Abstract] OR “MedDiet*”[Title/Abstract] OR “Mediterranean score*”[Title/Abstract] OR “MDS”[Title/Abstract] OR “aMED”[Title/Abstract])
Web of science	TS = (“Pancreatic Neoplasm*” OR “Cancer of Pancreas” OR “Cancer of the Pancreas” OR “Pancreatic Cancer*” OR “Pancreas Cancer*” OR “Pancreatic Carcinoma*” OR “Pancreatic Adenocarcinoma*”) AND TS = (“Mediterranean Diet*” OR “MedDiet*” OR “Mediterranean Dietary Pattern*” OR “Mediterranean Eating Pattern*” OR “Mediterranean Style Diet*”)
Embase	(“pancreatic cancer*” OR “pancreas cancer*” OR “pancreatic neoplasm*” OR “pancreas neoplasm*” OR “pancreatic tumor*” OR “pancreatic tumor*” OR “pancreas tumor*” OR “pancreas tumor*” OR “pancreatic carcinoma*” OR “pancreatic adenocarcinoma*”) AND (“Mediterranean diet*” OR “MedDiet*” OR “Mediterranean dietary pattern*” OR “Mediterranean eating pattern*” OR “Mediterranean style diet*”)
Cochrane library	(MeSH descriptor: [Pancreatic Neoplasms] explode all trees OR “pancreatic cancer*” OR “pancreas cancer*” OR “pancreatic neoplasm*” OR “pancreatic carcinoma*”) AND (MeSH descriptor: [Mediterranean Diet] explode all trees OR “Mediterranean diet*” OR “Mediterranean dietary pattern*” OR “MedDiet*”)

### 2.2 Inclusion and exclusion criteria

Two independent reviewers (YXZ and MYD) conducted a preliminary screening of titles and abstracts to identify eligible studies. Disagreements were resolved through discussion or consultation with a third reviewer (LYL). Full texts of potentially relevant articles were then reviewed against the following inclusion criteria: (1) Adults (≥18 years); (2) Observational studies (cohort or case-control) investigating the association between the MedDiet and pancreatic cancer; (3) Adherence to the MedDiet; (4) Comparison between patients with confirmed pancreatic cancer and a control population (non-pancreatic cancer); (5) Reported effect estimates, including Odds Ratios (OR), Relative Risks (RR), or Hazard Ratios (HR), with 95% Confidence Intervals (CI); (6) Published in English. Studies were included regardless of the specific definition of the MedDiet or geographical location, provided the exposure was explicitly labeled as such.

The exclusion criteria were as follows: (1) Population < 18 years of age; (2) Interventions focusing on other dietary patterns, supplements, single nutrients, or trace elements; (3) Outcomes lacking clear diagnostic criteria for pancreatic cancer or combining pancreatic cancer with other gastrointestinal malignancies; (4) Duplicate publications or studies utilizing overlapping datasets; (5) Meeting abstracts, reviews, case reports, editorials, or commentaries; (6) Studies lacking quantitative data (HR, RR, OR, and 95% CI).

### 2.3 Data extraction

**FIGURE 1:**
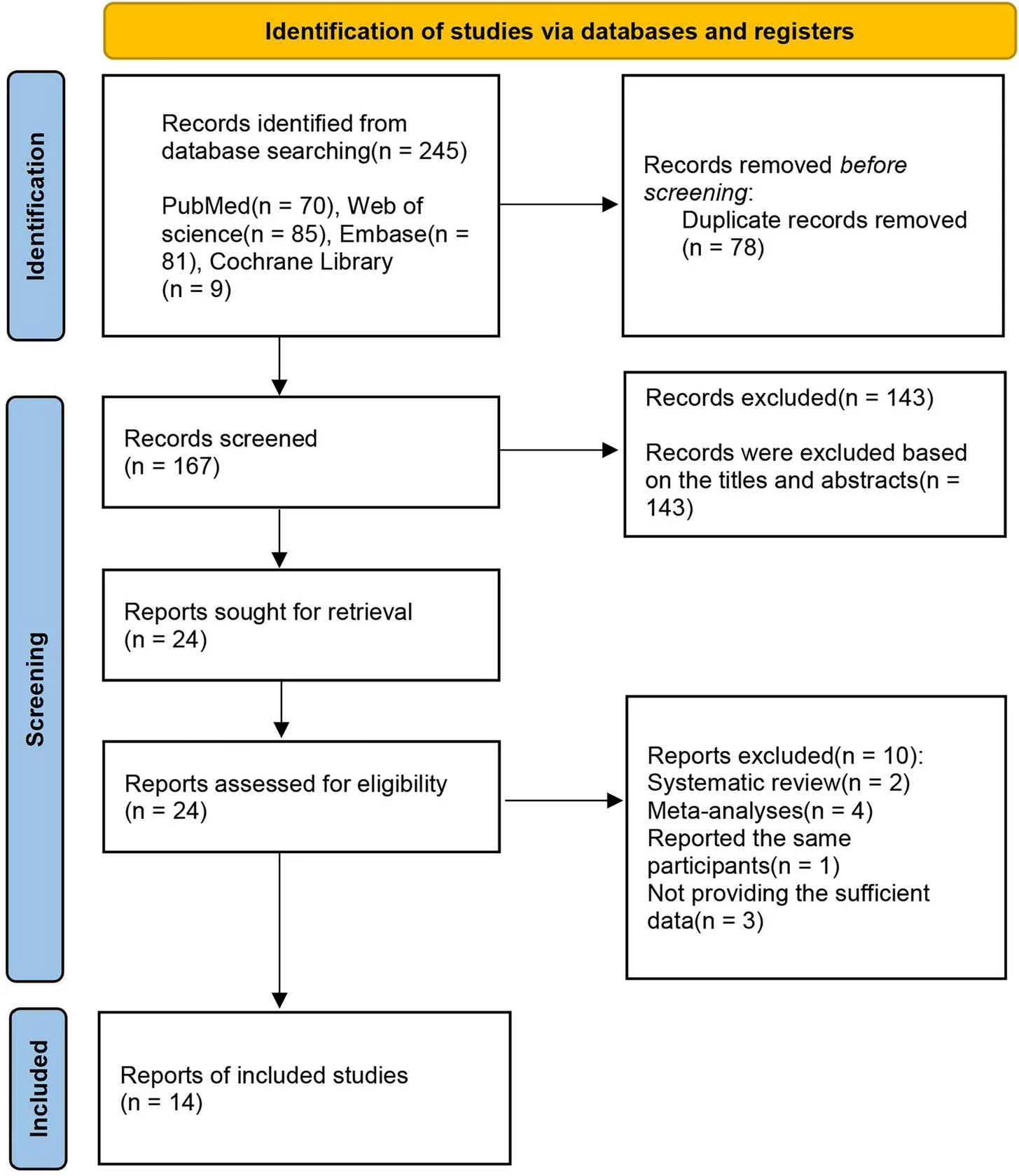
PRISMA flow diagram for new systematic reviews which included searches of data bases and registers only, the process of study selection.

Data were extracted using a standardized, predefined spreadsheet (Microsoft Excel 2019), consistent with previous methodologies (16, 17). The following variables were extracted from each eligible study: first author, publication year, country, study design, total population size, sex distribution, dietary assessment method (e.g., FFQ), MedDiet scoring system, effect estimates (HR/OR/RR) with 95% CIs, and study quality score (Supplementary Table 1). A PRISMA flow chart (Figure 1) was generated to document the study selection process.

### 2.4 Statistical analysis

Effect sizes (ES) were pooled using the reported HRs, RRs, or ORs along with their 95% CIs. Participants with the highest adherence to the MedDiet were compared to those with the lowest adherence. Heterogeneity among studies was assessed using the I^2^ statistic, with heterogeneity categorized as low (I^2^ < 25%), low-to-moderate (25% ≤ I^2^ < 50%), moderate-to-high (50% ≤ I^2^ ≤ 75%), or high (I^2^ > 75%).

To minimize potential bias arising from pooling different effect measures, we adopted the rare disease assumption proposed by Greenland and Thomas and further elaborated by Zhang and Yu (18, 19). Given that pancreatic cancer incidence was <5% in all included cohorts, ORs from case–control studies can be interpreted as approximations of RRs. Under this rare-disease assumption, we therefore pooled ORs and HRs to obtain a pooled relative effect estimate (12).

For continuous analyses, effect estimates were pooled according to the one-unit increase in the MedDiet adherence score as reported by each original study. Because the included studies used different MedDiet scoring systems with different score ranges and component definitions, a one-unit increase was interpreted as a within-study increment in the respective scoring system rather than as an identical absolute increase in MedDiet adherence across all scoring systems. Therefore, the pooled estimate should be interpreted as an overall association across heterogeneous scoring instruments rather than as a strictly standardized effect per equivalent unit of adherence.

Considering the anticipated heterogeneity arising from diverse geographic populations, varying MedDiet scoring systems, and different study designs, the random-effects model was exclusively employed to pool effect sizes. Potential publication bias was evaluated visually using funnel plots and statistically using Egger’s linear regression test (significance level *p* < 0.1000) (20). All analyses were performed using Stata 15.0 and RStudio version 4.6.1.

### 2.5 Sensitivity and subgroup analyses

To explore sources of heterogeneity and validate the robustness of the findings, sensitivity and subgroup analyses were conducted. Studies were stratified based on the type of variable reported (continuous vs. categorical). Subgroup analyses were performed by gender, regions, scoring method of the MedDiet and study design (cohort vs. case-control). Sensitivity analysis involved the sequential omission of individual studies to assess their impact on the pooled estimates.

## 3 Results

### 3.1 Literature search

The initial search yielded 245 records (PubMed: 70, Web of Science: 85, Embase: 81, Cochrane Library: 9). After removing 78 duplicates, 167 records remained. Title and abstract screening led to the exclusion of 143 records. Of the remaining 24 full-text articles assessed, 10 were excluded (reviews, overlapping datasets, or conference abstracts). Ultimately, 14 studies met the eligibility criteria (10, 11, 13, 14, 21–30) and were included in the meta-analysis. The selection process is detailed in Figure 1.

### 3.2 Study characteristics

The characteristics of the 14 included studies are summarized in Supplementary Table 1. Geographically, eight studies were conducted in Europe, four in the United States, one in Australia, and one in Singapore. All studies utilized Food Frequency Questionnaires (FFQs) to assess dietary intake, though they varied in validation status and the number of food items included. The analysis comprised 12 cohort studies and 2 case-control studies. Cohort studies generally reported Hazard Ratios (HR), while case-control studies reported Odds Ratios (OR).

### 3.3 Population characteristics

The cumulative sample size was substantial, with the two case-control studies alone contributing 978 (27) and 2,892 (24) participants, including 326 and 688 cases, respectively. Participants ranged in age from 18 to 86 years, with varying sex ratios across studies.

### 3.4 Mediterranean diet scoring

Various scoring systems were employed to quantify MedDiet adherence, including the Classic MedDiet Score (MDS), Alternative MedDiet Score (aMED), and others (aMDS, mMDS, arMED). Despite differences in specific components and cut-off points, all systems generally awarded positive points for high intake of beneficial foods (vegetables, fruits, whole grains, legumes, fish, olive oil) and negative points for detrimental foods (red and processed meats), typically adjusted for alcohol consumption.

### 3.5 Meta-analysis results

Given the moderate-to-high heterogeneity detected in the primary analyses, all pooled estimates reported in this section were derived exclusively from the random-effects model. Data were analyzed in two groups based on variable type: continuous (10 studies) and categorical (10 studies). Among these, four did not report eligible effect estimates for the overall population in the categorical analysis [specifically, Schulpen et al. (29) provided only sex-stratified data without a total sample estimate] and were thus excluded from the corresponding primary forest plot. One study (29) employed two scoring criteria. The version considered superior (mMED) by the original authors was selected for the analysis.

**FIGURE 2:**
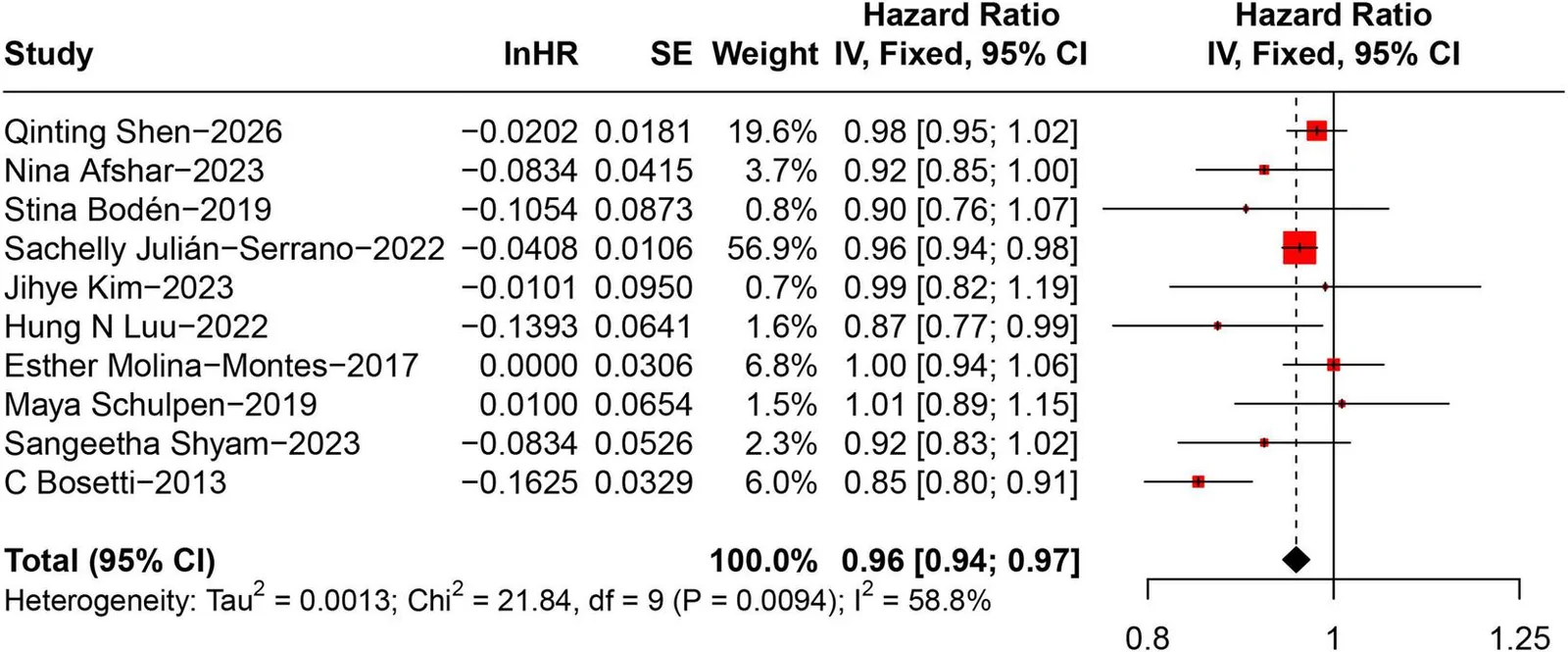
Forest plot of the association between adherence to the MedDiet (continuous variables) and pancreatic cancer risk.

**FIGURE 3:**
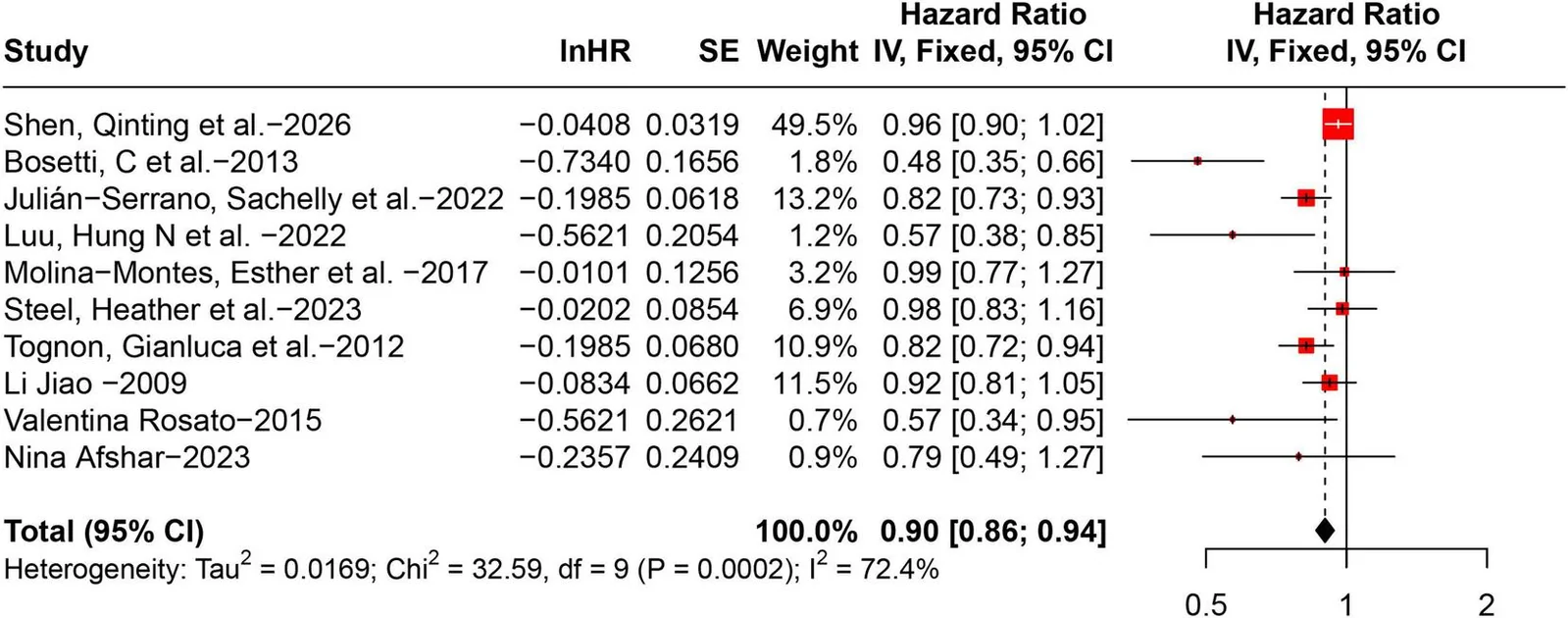
Forest plot of the association between adherence to the MedDiet (categorical variables) and pancreatic cancer risk.

The pooled relative effect estimates for a one-unit increase in the respective MedDiet adherence score were 0.96 (95% CI: 0.94–0.97, I^2^ = 58.8%, Figure 2), suggesting an association with a 4% lower risk per one-unit increase within the respective scoring system in adherence. Heterogeneity was moderate. Comparing the highest vs. lowest adherence groups, the pooled relative effect estimates were 0.90 (95% CI: 0.86–0.94, I^2^ = 72.4%, Figure 3), suggesting a 10% lower risk among the highest versus lowest adherence groups. Heterogeneity was moderate.

Visual inspection of the funnel plots suggested some asymmetry for both the continuous and categorical analyses (Supplementary Figures 1, 2). For continuous variables, although the funnel plot showed some asymmetry, Egger’s regression test did not detect statistically significant evidence of small-study effects (intercept = −0.767, *t* = −0.97, *P* = 0.362). In contrast, the funnel plot for categorical variables appeared asymmetric, and Egger’s regression test indicated statistically significant evidence of small-study effects (intercept = −2.175, *t* = −2.65, *P* = 0.029), suggesting potential publication bias (Supplementary Figures 3, 4). Given the limited number of included studies (*n* = 10 per analysis), which may reduce the statistical power of Egger’s test, and the presence of moderate-to-high heterogeneity, these findings should be interpreted with caution. However, we refrained from performing trim-and-fill correction because such adjustment may be unreliable with 10 or fewer studies per analysis.

Overall, the analysis consistently demonstrated that adherence to the MedDiet is significantly associated with a lower risk of pancreatic cancer.

### 3.6 Subgroup analyses

**FIGURE 4:**
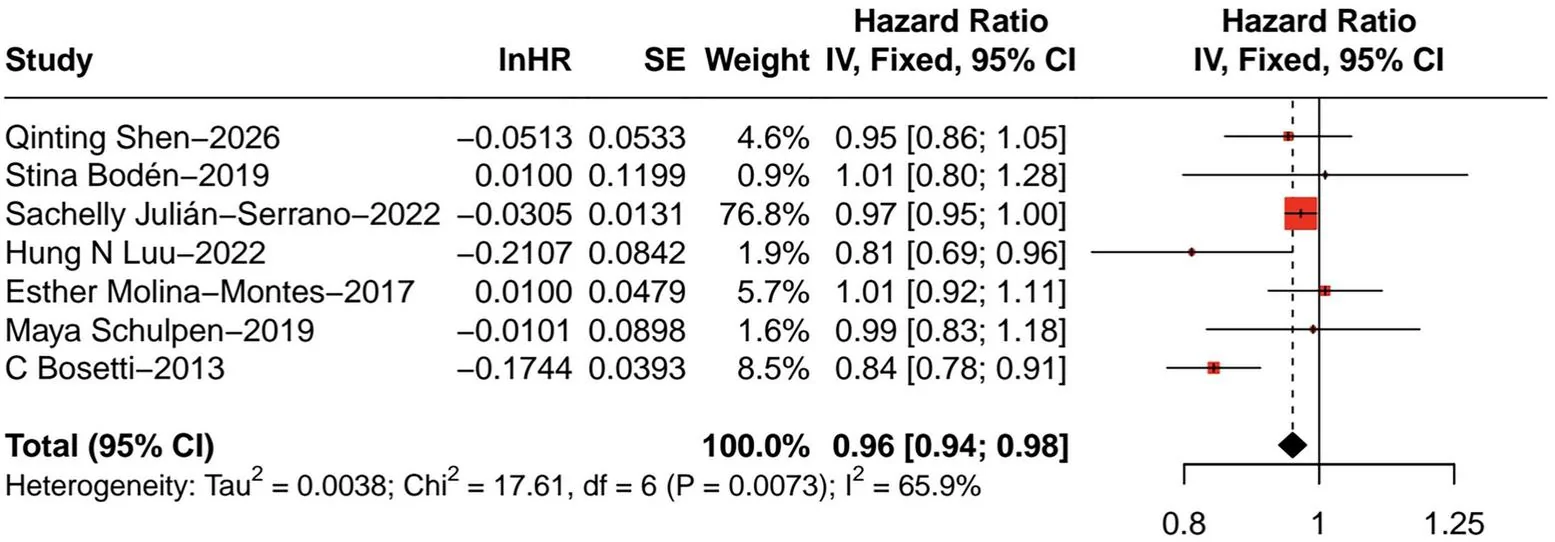
Forest plot of the association between adherence to the MedDiet (continuous variables) and pancreatic cancer risk in male.

**FIGURE 5:**
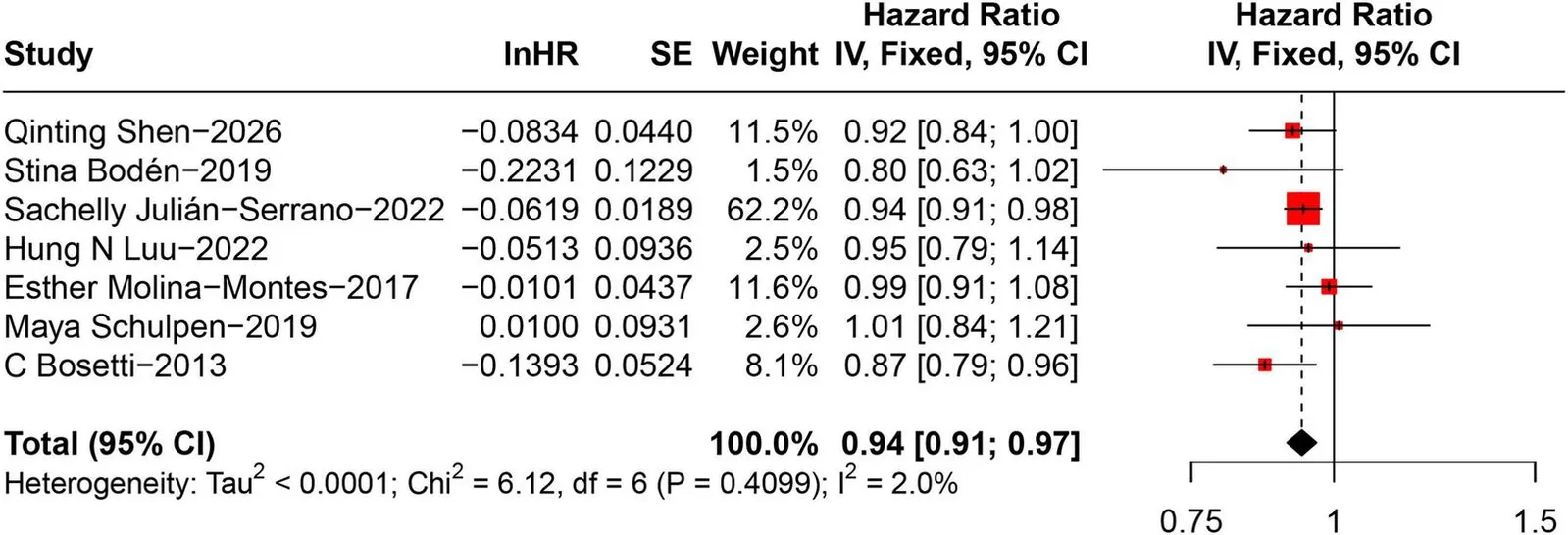
Forest plot of the association between adherence to the MedDiet (continuous variables) and pancreatic cancer risk in female.

**FIGURE 6:**
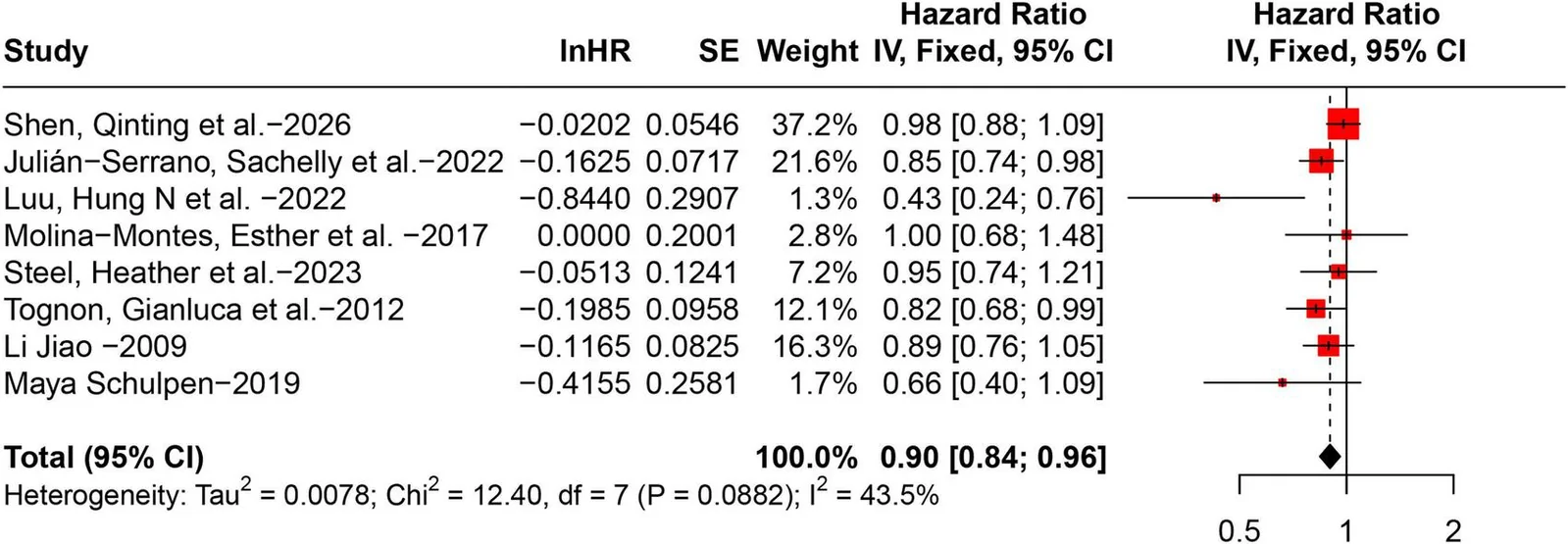
Forest plot of the association between adherence to the MedDiet (categorical variables) and pancreatic cancer risk in male.

**FIGURE 7:**
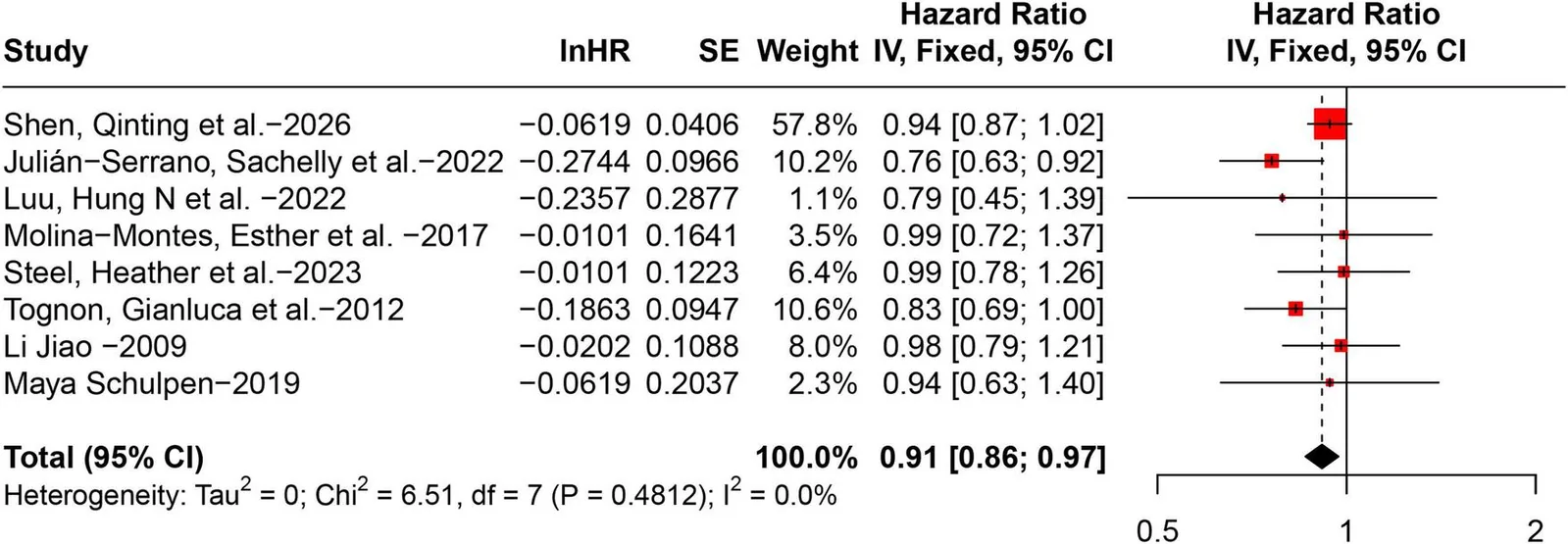
Forest plot of the association between adherence to the MedDiet (categorical variables) and pancreatic cancer risk in female.

In the continuous variable analysis, high MedDiet adherence was associated with a lower risk of PC in both males (pooled relative effect estimates = 0.96, 95% CI: 0.94–0.98, I^2^ = 65.9%, Figure 4) and females (pooled relative effect estimates = 0.94, 95% CI: 0.91–0.97, I^2^ = 2.0%, Figure 5). In the categorical analysis, similar inverse associations were consistently observed in males (pooled relative effect estimates = 0.90, 95% CI: 0.84–0.96, I^2^ = 43.5%, Figure 6) and females (pooled relative effect estimates = 0.91, 95% CI: 0.86–0.97, I^2^ = 0.0%, Figure 7), with no significant subgroup heterogeneity detected between sexes (*P*_*heterogeneity*_ = 0.4812).

When restricted to cohort studies, the inverse association remained significant for both continuous (HR = 0.96, 95% CI: 0.94–0.97, Supplementary Figure 5) and categorical variables (HR = 0.93, 95% CI: 0.88–0.97, Supplementary Figure 6).

Subgroup analyses were further performed stratified by MedDiet scoring systems, divided into classic MDS, aMED, and others.

In the continuous variable analysis, the pooled relative effect estimates were 0.88 (95% CI: 0.85–0.93, I^2^ = 31.7%, Supplementary Figure 9) for studies adopting classic MDS and 0.96 (95% CI: 0.95–0.98, I^2^ = 16.9%, Supplementary Figure 10) for studies using aMED, both indicating significant inverse associations. No significant association was observed among studies adopting others (HR = 0.99, 95% CI: 0.94–1.04, I^2^ = 0.0%, Supplementary Figure 11). In the categorical analysis, significant inverse associations were consistently detected across all scoring subgroups. The pooled relative effect estimates were 0.56 (95% CI: 0.45–0.72, I^2^ = 31.2%, Supplementary Figure 12) for classic MDS, 0.93 (95% CI: 0.88–0.97, I^2^ = 64.2%, Supplementary Figure 13) for aMED, and 0.86 (95% CI: 0.76–0.96, I^2^ = 42.5%, Supplementary Figure 14) for others.

Notably, the magnitude of beneficial association appeared strongest in studies utilizing classic MDS. Heterogeneity varied across scoring subgroups, suggesting that differences in MedDiet scoring criteria partially explained between-study heterogeneity.

Subgroup analyses stratified by geographic region were further performed separately for studies reporting continuous and categorical variables. Among studies with continuous variables, an inverse association between adherence to the MedDiet and pancreatic cancer risk was observed across Europe, the Americas, and other regions (Australia and Singapore). The pooled relative effect estimates were 0.96 (95% CI: 0.93–0.98, I^2^ = 73.0%, Supplementary Figure 15) for Europe, 0.96 (95% CI: 0.94–0.98, I^2^ = 0.0%, Supplementary Figure 16) for the Americas, and 0.90 (95% CI: 0.85–0.97, I^2^ = 0.0%, Supplementary Figure 17) for the other regions. Although the magnitude of the associations varied slightly among regions, the overall direction of the association remained consistent.

Similarly, among studies reporting categorical variables, adherence to the MedDiet was also associated with a reduced risk of pancreatic cancer in all geographic regions. The pooled relative effect estimates were 0.91 (95% CI: 0.87–0.96, I^2^ = 83.1%, Supplementary Figure 18) for Europe, 0.89 (95% CI: 0.82–0.96, I^2^ = 39.0%, Supplementary Figure 19) for the Americas, and 0.65 (95% CI: 0.48–0.89, I^2^ = 5.9%, Supplementary Figure 20) for the other regions.

Overall, the subgroup analyses demonstrated a consistent inverse association between adherence to the MedDiet and pancreatic cancer risk across all geographic regions in both continuous and categorical analyses.

### 3.7 Sensitivity analysis

Sensitivity analyses were conducted by sequentially excluding individual studies. For continuous variables, the pooled relative effect estimate remained stable between 0.91 and 0.98 (Supplementary Figure 7). For categorical variables, the pooled relative effect estimate fluctuated between 0.75 and 0.92 (Supplementary Figure 8). Crucially, no single study altered the direction of the association, supporting the robustness of the results.

### 3.8 Quality assessment

All included studies received NOS scores ≥ 7 and were considered to be of high methodological quality (10, 11, 13, 14, 21–30) (Table 2). However, GRADE rated the evidence as very low for both analyses: the continuous analysis was downgraded for heterogeneity (no publication bias), while the categorical analysis was downgraded for heterogeneity and potential publication bias (Supplementary Table 2).

**TABLE 2:** Newcastle-Ottawa scale score of the included studies.

Studies	1	2	3	4	5	6	7	8	9	Score
Juliàn-Serrano et al. (10)	0	1	1	1	1	1	1	1	1	8
Molina-Montes et al. (11)	1	1	1	1	1	1	1	1	0	8
Kim et al. (13)	1	1	1	1	1	1	1	0	0	7
Shyam et al. (14)	1	1	1	1	1	1	1	1	0	8
Afshar et al. (21)	1	1	1	1	1	1	1	1	1	9
Steel et al. (22)	1	1	1	1	1	1	1	1	0	8
Jiao et al. (23)	1	1	1	1	1	1	1	1	1	9
Bosetti et al. (24)	1	1	1	1	1	1	1	1	0	8
Luu et al. (25)	1	1	1	1	1	1	1	1	0	8
Tognon et al. (26)	1	1	1	1	1	1	1	0	0	7
Rosato et al. (27)	1	1	1	1	1	1	1	1	0	8
Bodèn et al. (28)	1	1	1	1	1	1	1	1	1	9
Schulpen et al. (29)	0	1	1	1	1	1	1	1	1	8
Shen et al. (30)	1	1	1	1	1	1	1	1	0	8

1, representativeness of the exposed cohort; 2, selection of the non-exposed cohort; 3, ascertainment of exposure; 4, outcome not present at baseline; 5, control for age and sex; 6, control for other confounding factors; 7, assessment of outcome; 8, enough long follow-up duration; 9, adequacy of follow-up of cohorts; aMED, alternate Mediterranean score.

## 4 Discussion

In this systematic review and meta-analysis, we investigated the association between adherence to the MedDiet and the risk of pancreatic cancer (PC) by integrating data from existing observational studies. The results suggest that a high-adherence MedDiet pattern shows a significant inverse association with pancreatic cancers.

Our findings consistently demonstrate an inverse association between MedDiet adherence and pancreatic cancer risk. This aligns with results from several large prospective cohort studies. For instance, Trichopoulou et al. (31) originally highlighted the association of this dietary pattern on overall mortality. More recently, Schwingshackl and Hoffmann. (32) reported that higher adherence to the MedDiet was associated with a lower risk of aerodigestive cancers, although no significant association was observed for pancreatic cancer.

A key strength of our analysis is the categorization of the variables. The analysis of continuous variables (10 studies) indicated that each unit increase in MedDiet adherence was associated with a 4% lower risk of pancreatic cancer. This trend was further corroborated by the categorical analysis, where the highest adherence group showed a substantial 10% lower risk compared to the lowest adherence group. This strengthened inverse association in the categorical analysis underscores the association of strict adherence, widening the risk disparity between high and low compliance groups. Collectively, this evidence supports a potential inverse association, consistent with the relevance reported in the 2017 systematic review by Schwingshackl et al. (33).

Furthermore, sensitivity analyses confirmed the robustness of our core conclusions. In the continuous variable analysis, sequentially eliminating individual studies resulted in pooled relative effect estimates remaining within a narrow range and consistently below 1. This indicates that the inverse association is reliable and not driven by any single study, regardless of fluctuations in individual datasets.

Despite the overall inverse association, findings across individual studies have not been entirely consistent. Notably, the European Prospective Investigation into Cancer and Nutrition (EPIC) study found no significant association. Several factors may contribute to these discrepancies. First, variations in study design—particularly differences in sample sizes and the number of cases—may result in insufficient statistical power to detect modest inverse associations. Second, population heterogeneity plays a role; the inverse association may be more pronounced in traditional Mediterranean populations (e.g., Italy) compared to non-Mediterranean populations (e.g., the United States), where the effect might be diluted by differences in genetic background, lifestyle, and baseline dietary patterns.

Subgroup analyses provided further insight into these variations. Although the magnitude of the inverse association varied slightly by gender, both groups showed significantly lower risk. Notably, the female subgroup exhibited extremely low heterogeneity in the categorical analysis, indicating a relatively consistent association across the included studies. However, this finding should be interpreted cautiously and should not be taken as evidence of a causal effect or greater benefit of MedDiet adherence among women. Additionally, separate analysis of high-quality prospective cohort studies reconfirmed significant associations. As prospective studies establish the temporal sequence of exposure preceding outcome, this provides stronger evidence for a potential inverse association. Stratified analyses by scoring systems revealed significant inverse associations for classic MDS and aMED in continuous models, whereas the association was non-significant for others. All scoring subgroups showed beneficial association in categorical analyses, with the strongest inverse association observed for classic MDS. Differences in scoring criteria for core food components may account for variable effect sizes, suggesting that the choice of MedDiet assessment tool can influence risk estimates. After stratification by geographic region, a consistent inverse association between MedDiet adherence and pancreatic cancer risk was observed in both continuous and categorical variable analyses. Although the effect estimates varied slightly across regions, the direction of the association remained consistently negative. The other regions (Australia and Singapore) showed a more pronounced inverse trend, which might be attributable to the relatively small number of studies included from these regions. Noticeable regional differences in statistical heterogeneity were observed (I^2^ = 83% in Europe, 39% in the Americas, and 5% in other regions). These disparities may be largely explained by variations in genetic backgrounds, baseline lifestyles, habitual dietary patterns, and the application of Mediterranean Diet scoring tools, as well as differences in sample sizes and the number of included studies across subgroups. Indeed, in regions with a larger number of included studies, the heterogeneity was significantly greater than in other regions. Overall, the association exhibited good consistency across different geographic settings, suggesting that the potential protective effect of MedDiet against pancreatic cancer may not be limited by regional characteristics and supports favorable robustness for external generalizability.

Compared with Nucci D. et al.’s meta-analysis (12), our study strengthens evidence for the MedDiet and pancreatic cancer association in three ways. First, we extended literature searches up to July 2026 and included five newly-published studies from 2023 to 2026, enlarging the overall sample size and improving statistical power. Second, our analytical plan is more rigorous. Besides conventional categorical comparisons, we performed linear continuous-variable analysis and found a one-unit increment corresponded to a 4% lower risk in MedDiet score. We assessed publication bias separately and validated outcomes using cohort only analysis. Third, we identified low heterogeneity among females. Additional subgroup analyses by sex, geographic region, scoring system, and study design provided further insight into the consistency of the observed associations, although these analyses should be interpreted as exploratory given the very low certainty of evidence and the limited number of studies in several subgroups.

Several biological pathways may explain the observed inverse association, although these remain speculative given the observational nature of the current evidence. The MedDiet is rich in antioxidants and omega-3 fatty acids, which can neutralize free radicals and mitigate chronic inflammation—a known driver of pancreatic carcinogenesis (34, 35). Additionally, the diet’s high fiber content and low glycemic load improve insulin sensitivity, potentially reducing chronic hyperinsulinemia (36, 37). Finally, the synergistic effects of MedDiet components may support gut microbial diversity and systemic immune homeostasis, further contributing to its anti-tumor potential (38, 39). Together, these pathways provide a plausible biological rationale for the 10% lower risk observed in our analysis.

This study also has significant limitations. An important methodological limitation concerns the comparability of continuous MedDiet scores across studies. The included studies used different scoring systems, including MDS, aMED, aMDS, mMDS, and arMED, which differed in score ranges, component definitions, and scoring criteria. Consequently, a one-unit increase in one scoring system does not necessarily represent the same absolute change in MedDiet adherence as a one-unit increase in another scoring system. In the present meta-analysis, continuous effect estimates were therefore interpreted as associations per one-unit increase within the respective scoring system rather than as a universally equivalent unit of MedDiet adherence. Although this approach allowed the available continuous estimates to be synthesized, residual heterogeneity arising from differences in score construction may have influenced the pooled estimate. The subgroup analyses by scoring system provided additional assessment of the consistency of the association across different scoring instruments, as noted by Schwingshackl et al. (40), variations in MedDiet Scores (MDS) pose a challenge. Included studies used various scoring systems (MDS, aMED, aMDS, etc.) with differing definitions and standards for components like alcohol, whole grains, and dairy. Furthermore, food definitions vary geographically; for instance, “lipid intake” may refer to extra virgin olive oil in Southern Europe (27), but vegetable oils in Asia (28) or be quantified by MUFA/SFA ratios in Northern Europe (29). Additionally, approximately 90% of studies used population-specific median scoring. While this avoids cultural bias, it reflects relative rather than absolute adherence, hindering direct cross-study comparisons of Hazard Ratios. In addition, conducting multiple subgroup analyses across gender, geographic regions, dietary scoring systems, and study designs increases the risk of Type I errors (false positives). Because formal statistical adjustments for multiple comparisons (such as Bonferroni or False Discovery Rate controls) were not applied, findings from these stratified analyses should be interpreted with appropriate caution as exploratory rather than definitive. Finally, only the categorical analysis suggested publication bias, whereas the continuous analysis did not.

Another analytical limitation is that we could not conduct formal dose-response meta-analysis. Most included studies only provided risk estimates for a single per-unit increase in MedDiet score or a simple comparison between the highest and lowest adherence groups, without stratified case and population data across three or more adherence tiers. Such multi-level data are required to fully assess linear or non-linear relationships between diet adherence and pancreatic cancer risk (41). Accordingly, we can only report a simple inverse association between cancer risk and higher adherence, rather than drawing robust conclusions about comprehensive graded risk patterns. Therefore, future studies with multiple quantitative exposure categories are warranted to perform a formal dose-response meta-analysis.

In addition, moderate heterogeneity was detected across both continuous and categorical pooled analyses. Although subgroup stratifications were conducted by gender, regions, scoring method of the MedDiet, and study design, we lacked sufficient stratified raw data to perform further analyses for other critical potential confounders. Specifically, we could not conduct stratified analyses by follow-up duration or study quality scores.

The results of this study were compared with those of several recent authoritative reviews. Although the updated study by Morze et al. (42) and the umbrella review by Gianfredi et al. (43) had indicated that there was no significant association between the MedDiet and the risk of pancreatic cancer, in 2023, Nucci D. et al. (12) conducted a latest analysis of 1,301,320 participants and found that a high adherence to the MedDiet could significantly reduce the risk of pancreatic cancer by 18%. The conclusion of this study is highly consistent with the findings of Nucci D. et al. The main reason for the difference in these conclusions lies in the fact that the latest research has included more recent high-quality studies, which compensates for the insufficient statistical power of the earlier meta-analysis due to the limited number of included studies (for example, the early studies only included four articles). Furthermore, this further indicates the view of Gianfredi et al. that a diet dominated by plants is beneficial for preventing pancreatic cancer.

The findings of this meta-analysis suggest an inverse association between adherence to the MedDiet and pancreatic cancer risk; however, the certainty of the evidence was rated as very low for both continuous and categorical analyses according to the GRADE framework. Therefore, these findings should be interpreted cautiously and should not be considered sufficient to establish a causal protective effect of the MedDiet against pancreatic cancer. Although the observed association may provide supportive evidence for further investigation of dietary patterns in pancreatic cancer prevention, it does not by itself justify specific public health recommendations to promote the MedDiet for pancreatic cancer prevention. Future prospective cohort studies and, where feasible, well-designed dietary intervention studies are needed to determine whether improving adherence to the MedDiet can reduce pancreatic cancer risk. Although the female subgroup showed low statistical heterogeneity, this finding should be considered exploratory and should not be interpreted as evidence that interventions targeting women would necessarily result in favorable outcomes. Future research should further investigate potential sex-specific associations using adequately powered and prospectively designed studies.

## 5 Conclusion

This systematic review and meta-analysis suggests an inverse association between higher adherence to the MedDiet and pancreatic cancer risk. However, the certainty of the evidence was rated as very low according to the GRADE framework, and the observational nature of the included studies precludes firm conclusions regarding causality. Therefore, the findings should be interpreted cautiously and considered hypothesis-generating rather than sufficient to support definitive dietary recommendations for pancreatic cancer prevention. Future well-designed prospective studies and dietary intervention trials are warranted to clarify the potential protective association and establish its clinical and public health relevance.

## Data Availability

The original contributions presented in this study are included in this article/Supplementary material, further inquiries can be directed to the corresponding author.
