# Supplementary material for: Association between adherence to the Mediterranean Diet and pancreatic cancer risk: a systematic review and meta-analysis
Source: Front Nutr. 2026 Sep 4;13:1925019. doi: 10.3389/fnut.2026.1925019 (PMC13585576; doi:10.3389/fnut.2026.1925019)
Supplement: Supplementary file 1 [file Supplementary_file_1.doc]

***Supplementary Material***

Table S1 Original data

Table S2 GRADE assessment of the certainty of evidence

Figure S1 Continuous variable funnel plot

Figure S2 Categorical variable funnel plot

Figure S3 Continuous variable egger’s test

Figure S4 Categorical variable egger’s test

Figure S5 Continuous variable cohorts studies forest plots

Figure S6 Categorical variable cohorts studies forest plots

Figure S7 Continuous variable sensitivity analysis

Figure S8 Categorical variable sensitivity analysis

Figure S9 Continuous variable MDS forest plots

Figure S10 Continuous variable aMED forest plots

Figure S11 Continuous variable other scoring methods forest plots

Figure S12 Categorical variable MDS forest plots

Figure S13 Categorical variable aMED forest plots

Figure S14 Categorical variable other scoring methods forest plots

Figure S15 Continuous variable Europe forest plots

Figure S16 Continuous variable Americas forest plots

Figure S17 Continuous variable other regions forest plots

Figure S18 Categorical variable Europe forest plots

Figure S19 Categorical variable Americas forest plots

Figure S20 Categorical variable other regions forest plot

**Table S1 Original data**

| **Author, Year [Ref.]** | **Study Design** | **Total** | **Gender** | **Categorical variable effect size** | **Continuous variable effect size** | **Nation** | **Mediterranean Score Used** | **Tool Used for Dietary Assessment** | **Adjusted covariates** | **NOS** |
| --- | --- | --- | --- | --- | --- | --- | --- | --- | --- | --- |
|  |  | **Sample** |  |  |  |  |  |  |  |  |
| Shen, Qinting,2026 | CO | Ca:10748 | Ca: F = 5757 | Total sample: HR = 0.96 (0.90 – 1.02) | Total sample: HR = 0.98 (0.95 – 1.01) | Europe | aMED,maMED | FFQ | BMI, smoking, personal history of diabetes, energy intake | 9 |
|  |  | Co: 2315406 | Co: F =NA | M:HR=0.98 (0.88–1.09) | M:HR=0.98 (0.95–1.02) |  |  |  |  |  |
|  |  |  |  | F:HR=0.94 (0.87–1.02) | F:HR=0.98 (0.95–1.01) |  |  |  |  |  |
| Nina Afshar,2023 | CO | Ca: 258 | Ca: F = 142 | Total sample: HR = 0.79 (0.49 – 1.26) | Total sample: HR = 0.92 (0.85 – 1.00) | Australia | MDS | FFQ | sex, height, country of birth, education, socio-economic position, PA, energy intake, smoking status, pack-years smoking, years since quitting smoking, and alcohol intake | 9 |
|  |  | Co: 33,690 | Co: F = 20,407 |  |  |  |  |  |  |  |
| Stina Bodén,2019 | CO | Ca: 223 | Ca: F = 107 | NA | Total sample: HR = 0.90 (0.76–1.07) | Europe | aMDS | FFQs | EI, BMI, smoking, PA, and education | 8 |
|  |  | Co: 100,881 | Co: F = 51,001 |  | M: HR = 1.01 (0.80–1.28) |  |  |  |  |  |
|  |  |  |  |  | F: HR = 0.80 (0.63–1.02) |  |  |  |  |  |
| Bosetti, C,2013 | CC | Ca: 688 | Ca: F = 285 | Total sample: OR = 0.48 (0.35–0.67) | Total sample: OR =0.85（0.80-0.91） | Europe | MDS,MEPI,MAI | structured questionnaire, simplified dietary section and FFQ | centre, age, sex, year of interview, education, BMI, smoking, alcohol, history of T2D | 9 |
|  |  | Ct: 2,204 | Co: F = 715 |  | M: OR = 0.84 (0.78–0.91) |  |  |  |  |  |
|  |  |  |  |  | F: OR = 0.87 (0.79–0.97) |  |  |  |  |  |
| Li Jiao,2009 | CO | Ca: 1,057 | Ca: F = 382 | Total sample: RR = 0.92 (0.81–1.05) | NA | America | aMED | FFQ | Sex, age, race, educational level, marital status, total energy intake, smoking status, alcohol use, dietary quality, BMI and PA. | 9 |
|  |  | Co: 450,416 | Co: F = 187,018 | M:RR=0.89 (0.76–1.05) |  |  |  |  |  |  |
|  |  |  |  | F: RR = 0.98 (0.79–1.21) |  |  |  |  |  |  |
| Julián-Serrano,2022 | CO | Ca: 3,137 | Ca: F = n.a. | Total sample: HR = 0.82 (0.73–0.93) | Total sample: HR = 0.96 (0.94–0.98) | America | aMED | FFQ | age at baseline, sex , smoking, BMI, T2D, EI | 9 |
|  |  | Co: 535,824 | Co: F = 220,044 | M: HR = 0.85 (0.74–0.98) | M: HR = 0.97 (0.95–1.00) |  |  |  |  |  |
|  |  |  |  | F: HR = 0.76 (0.63–0.92) | F: HR = 0.94 (0.91–0.98) |  |  |  |  |  |
| Jihye Kim,2023 | CO | Ca: 951 | Ca: F = n.a. | NA | Total sample: HR = 0.99 (0.82, 1.19) | America | aMED | FFQ | NA | 8 |
|  |  | Co: 213,002 | Co: F = 166,728 |  |  |  |  |  |  |  |
| Luu, Hung N,2021 | CO | Ca: 311 | Ca: F = 149 | Total sample: HR = 0.57 (0.38–0.85) | Total sample: HR = 0.87 (0.77–0.99) | Singapore | aMED | FFQ | age, sex , dialect, year of enrolment, education, smoking, smoking pack-years, coffee drinking, EI, BMI, T2D | 9 |
|  |  | Co: 61,321 | Co: F = NA | M: HR = 0.43 (0.24–0.75) | M: HR = 0.81 (0.69–0.96) |  |  |  |  |  |
|  |  |  |  | F: HR = 0.79 (0.45–1.39) | F: HR = 0.95 (0.79–1.14) |  |  |  |  |  |
| Molina-Montes,2017 | CO | Ca: 865 | Ca: F = 469 | Total sample: HR = 0.99 (0.77–1.26) | Total sample: HR = 1.00 (0.94–1.06) | Europe | arMED | FFQ and diet history | EI, BMI, smoking status and intensity, alcohol, T2D | 9 |
|  |  | Co: 477,309 | Co: F = 335,060 | M: HR = 1.00 (0.68–1.49) | M: HR = 1.01 (0.92–1.11) |  |  |  |  |  |
|  |  |  |  | F: HR = 0.99 (0.72–1.37) | F: HR = 0.99 (0.91–1.08) |  |  |  |  |  |
| Valentina Rosato,2015 | CC | Ca: 326 | Ca: F = 152 | Total sample: OR = 0.57 (0.34–0.95) | NA | Europe | MDS | FFQ | sex, age, study centre, education, BMI, smoking, alcohol, T2D, EI | 8 |
|  |  | Ct: 652 | Ct: F = 304 |  |  |  |  |  |  |  |
| Maya Schulpen,2019 | CO | Ca: 793 | Ca: F = 415 | mMDS: M: HR = 0.66 (0.40–1.10) | mMDS: Total sample: HR = 1.01 (0.89–1.15) | Europe | aMED,mMED | FFQ | age, smoking status, smoking frequency, smoking duration, BMI, EI, alcohol, T2D, family history of PC, education, nonoccupational PA | 9 |
|  |  | Co: 62,573 | Co: F = 29,751 | F: HR = 0.94 (0.63–1.40) | M: HR = 0.99 (0.83–1.18) |  |  |  |  |  |
|  |  |  |  |  | F: HR = 1.01 (0.84–1.21) |  |  |  |  |  |
| Sangeetha Shyam,2023 | CO | Ca: 136 | all F | NA | Total sample: HR = 0.92 (0.83-1.02） | Europe | MDS | FFQ | age, smoking status, education, PA | 9 |
|  |  | Co: 35,365 |  |  |  |  |  |  |  |  |
| Steel, Heather,2023 | CO | Ca: 1,779 | Ca: F = 950 | Total sample: HR = 0.98(0.83-1.16) | NA | America | aMED | FFQ | age, sex, race/ethnicity, education, diabetes, family history of PC, PA, smoking variables, total energy intake, BMI and alcohol consumption | 9 |
|  |  | Co: 177,313 | Co: F = 95,905 | M:HR= 0.95 (0.75, 1.22) |  |  |  |  |  |  |
|  |  |  |  | F: HR = 0.99 (0.78, 1.26) |  |  |  |  |  |  |
| Tognon, Gianluca,2012 | CO | Ca: 92 | Ca: F = 45 | Total sample: HR = 0.82 (0.72–0.94) | NA | Europe | mMDS | FFQ | age, obesity, smoking status, education, PA | 9 |
|  |  | Co: 77,151 | Co: F = 39,605 | M: HR = 0.82 (0.68–0.99) |  |  |  |  |  |  |
|  |  |  |  | F: HR = 0.83 (0.69–1.00) |  |  |  |  |  |  |

**Table S2: GRADE assessment of the certainty of evidence**

| **Outcomes** | **No. of Studies (Design)** | **Risk of Bias** | **Inconsistency** | **Indirectness** | **Imprecision** | **Publication Bias** | **Upgrade Factors** | **Certainty** |
| --- | --- | --- | --- | --- | --- | --- | --- | --- |
| Pancreatic cancer incidence (highest vs. lowest adherence) | 10 studies (8 cohorts + 2 case-controls studies) | Not serious | Serious (substantial heterogeneity, *I*²=72.4%) | Not serious | Not serious | Serious | None | Very Low |
| Pancreatic cancer incidence  (per 1-point increase in adherence score) | 10 studies (10 cohorts studies) | Not serious | Serious (substantial heterogeneity, *I*²=58.8%) | Not serious | Not serious | Not serious | None | Very Low |

**NOS**, Newcastle–Ottawa Scale; ***I*²**, Higgins heterogeneity statistic. According to the GRADE approach, evidence from observational studies starts at low certainty and may be downgraded or upgraded based on predefined criteria.

**Figure S1 Continuous variable funnel plot**

**Figure S2 Categorical variable funnel plot**

**Figure S3 Continuous variable egger’s test**

**Figure S4 Categorical variable egger’s test**

**Figure S5 Continuous variable cohorts studies forest plots**

**Figure S6 Categorical variable cohorts studies forest plots**

**Figure S7 Continuous variable sensitivity analysis**

**Figure S8 Categorical variable sensitivity analysis**

**Figure S9 Continuous variable MDS forest plots**

**Figure S10 Continuous variable aMED forest plots**

**Figure S11 Continuous variable other scoring methods forest plots**

**Figure S12 Categorical variable MDS forest plots**

**Figure S13 Categorical variable aMED forest plots**

**Figure S14 Categorical variable other scoring methods forest plots**

**Figure S15 Continuous variable Europe forest plots**

**Figure S16 Continuous variable Americas forest plots**

**Figure S17 Continuous variable other regions forest plots**

**Figure S18 Categorical variable Europe forest plots**

**Figure S19 Categorical variable Americas forest plots**

**Figure S20 Categorical variable other regions forest plots**
